# Culture method for promoting efficient ciliary growth in the *Chlamydomonas* *pf23* mutant defective in dynein preassembly

**DOI:** 10.17912/micropub.biology.002069

**Published:** 2026-04-14

**Authors:** Ryosuke Yamamoto, Takahide Kon

**Affiliations:** 1 Department of Biological Sciences, Graduate School of Science, Osaka University, Osaka, Ōsaka, Japan

## Abstract

*Chlamydomonas reinhardtii* is an ideal model organism for studying the cytoplasmic preassembly of ciliary dyneins. However, under normal liquid-culture conditions, *Chlamydomonas* preassembly-deficient mutants often show no cilia or a small number of ciliated cells (i.e., low ciliation ratio), which hinders further analysis of both ciliary dyneins and the phenotypes of these mutants. In this brief report, we present a modified culture method for one of the *Chlamydomonas* preassembly-deficient mutants, *pf23* . This method enables researchers to obtain enough *pf23* cilia for small-scale biochemical, biophysical, structural and phenotypic analyses.

**Figure 1. Culture method for the Chlamydomonas pf23 mutant with difficulty growing cilia:** This is a schematic illustration of the ciliation method for *pf23* , the *Chlamydomonas* preassembly-deficient mutant. The panels [( **A** ) - ( **F** )] in this figure are explained in the " **Methods** " section of the main text. In our laboratory, we check the ciliary growth of the *pf23* cells transferred to the SG medium every day [panel ( **E** )]. As early as 1 day after transferring the cells, we often observed that they had grown sufficient cilia to perform small-scale experiments. Panel ( **E** ) also shows a ciliated *pf23* cell using this method as an inset. This figure was created using PowerPoint (Microsoft).

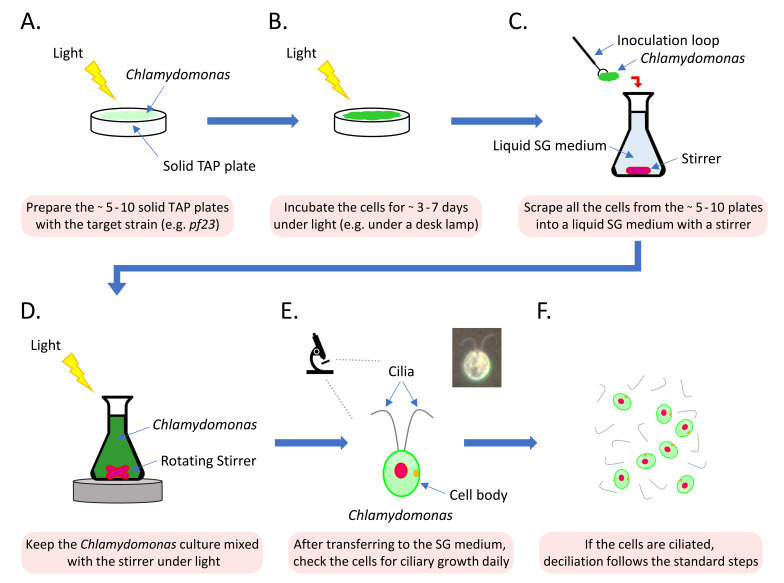



## Description

*Chlamydomonas* is an excellent model organism (Marshall, 2024; Ostrowski et al., 2011; Vincensini et al., 2011) for studying the composition and function of motile cilia, organelles that play vital roles in various important biological processes in eukaryotes, such as fertility and development (Satir & Christensen, 2007). In this regard, this tiny alga serves as an outstanding organism for studying the pathogenesis of primary ciliary dyskinesia (PCD), a human disease caused by deficient ciliary motility (Chodhari et al., 2004; Zariwala et al., 2011). Motile cilia are powered by motor-protein complexes called "ciliary dyneins" (Kamiya & Yagi, 2014; King et al., 2023; Yamamoto et al., 2021). The ciliary dyneins are preassembled in the cytoplasm before being transported into cilia (in a sequential process called "preassembly")(Desai et al., 2017; Mitchell & Yamamoto, 2023), and defects in this process also cause PCD in humans [e.g. (Mitchison et al., 2012; Omran et al., 2008; Tarkar et al., 2013)]. Across species, many *Chlamydomonas* preassembly-deficient mutants have also been identified [e.g. (Dean & Mitchell, 2013; Desai et al., 2015; Huang et al., 1979; Kamiya, 1988; Yamamoto et al., 2010)]. However, researchers often face a major challenge when studying the mutants: under the standard liquid tris-acetate-phosphate (TAP) medium condition (Gorman & Levine, 1965; Harris, 1989), these preassembly mutants often grow no or very short cilia, which are insufficient for various experimental analyses. Through testing several culture conditions, we found that, under a specific condition, a classical preassembly mutant, *pf23* (Huang et al., 1979), tends to grow cilia more efficiently than under the standard liquid TAP medium condition. In this brief report, we describe a simple culture method that results in efficient ciliary growth in the *pf23* mutant and provides enough cilia for small-scale biochemical, biophysical, structural and phenotypic analyses. In our laboratory, we mainly use this method, or modified versions of it, to isolate cilia from *pf23* .

**Methods**

The protocol for our new culture method ( **Figure 1** ) is described as follows:

[0] (Before the experiment/culture) Make ~ 200 - 500 mL of the liquid SG [Sager and Granick, or minimal (M)] medium [originally reported in (Sager & Granick, 1953), see also (Harris, 1989) and the *Chlamydomonas* resource center (https://www.chlamycollection.org/) for basic/conventional compositions] in a conical flask. Place a magnetic stirrer in the SG medium, then autoclave the SG medium and cool it to room temperature. There is no need to prepare bubbling equipment for aeration.

[1] Inoculate the target strain (in this case, *pf23* ) onto the ~ 5 - 10 solid TAP plates (Gorman & Levine, 1965; Harris, 1989), each measuring ~ 90 mm, and ensure the *Chlamydomonas* cells are distributed evenly across the plates ( **Figure 1A** ). Incubate the cells at room temperature under appropriate light (e.g. under a desk lamp) for ~ 3 - 7 days to allow them to grow well and turn the plates green ( **Figure 1B** ).

[2] Using an inoculation loop, scrape all the cells (cultured for ~ 3 - 7 days) from all the solid TAP plates and transfer them into the ~ 200 - 500 mL of the liquid SG medium (prepared in [0])( **Figure 1C** ). Since all the cells from the ~ 5 - 10 solid TAP plates are in the liquid SG medium, the medium should be green to dark green.

[3] Gently mix the *Chlamydomonas* culture in the SG medium ( **Figure 1D** ) at room temperature under appropriate light (e.g. under a desk lamp) with the magnetic stirrer (placed in the SG medium in [0]). There is no need for bubbling/aeration. The stirring should be moderately strong enough to disrupt the mother cell walls of palmelloid cells (Iwasa & Murakami, 1968; Khona et al., 2016; Wingfield & Lechtreck, 2018) without disrupting the cells themselves.

[4] Keep the *Chlamydomonas* culture mixed in the SG medium with the stirrer under light. The cells will often grow cilia 1 - 3 days after being transferred to the liquid SG medium. Using conventional light microscopy (e.g. Olympus BX50, at ~ ×200 magnification), check if the cells are ciliated every day after transferring them to the SG medium ( **Figure 1E** ). Once the cells are sufficiently ciliated (e.g. ciliation ratio ≧ ~ 70%), deciliate them ( **Figure 1F** ) and collect the cilia using the standard procedure (Craige et al., 2013; Witman, 1986).

Note that all the procedures should be aseptic, and contamination of the *Chlamydomonas* cultures by bacteria or fungi, especially during mixing in the SG medium, should be avoided. With this method, one of the *Chlamydomonas* preassembly-deficient mutants, *pf23* , tends to grow cilia efficiently. To demonstrate its effectiveness, we cultured *pf23* using this method ( **Figure 1E** ) and conducted an empirical evaluation of ciliation for this brief report. The SG medium used was essentially identical to the medium (https://chlamycollection.org/SG.html) listed at the *Chlamydomonas* resource center, with only minor modifications. 1 day after transferring to the SG medium, the ciliation ratio of *pf23* was approximately ≧ ~ 80%, based on a rough visual estimation. In the past, researchers observed that some preassembly-deficient mutants tend to grow cilia at a late culture stage in the nutrient-rich media (including the TAP medium)[e.g. (Huang et al., 1979; Yamamoto et al., 2010)], i.e., when nutrients in the nutrient-rich media decrease from the initial high amounts. We incorporated this observation into this method, establishing an easy way to grow *pf23* cilia. Due to the availability of reagents/chemicals as well as historical reasons, compositions of the SG medium somewhat vary between laboratories studying *Chlamydomonas* , but we believe most of these media would work well for this method. In addition, in this method, other nutrient-poor *Chlamydomonas* media [e.g. nitrate-as-nitrogen-source (N) medium (Sager & Granick, 1953), see also the *Chlamydomonas* resource center (https://www.chlamycollection.org/)] could potentially be used as an alternative to the SG medium.

**Conclusion**

We have long used this method, or modified versions of it [e.g. usage of "Hutner’s trace elements" (Hutner et al., 1950) instead of the trace elements], to isolate sufficient amounts of cilia from *pf23* , the classical *Chlamydomonas* preassembly-deficient mutant. This method is simple and convenient, can be performed using standard equipment, and is workable in most laboratories studying *Chlamydomonas* . This method could also be applicable to other *Chlamydomonas* ciliary and/or preassembly-deficient mutants that grow very short or few cilia under the standard TAP medium condition, such as *pf13* (Huang et al., 1979; Omran et al., 2008) and *pf22* (Huang et al., 1979; Mitchison et al., 2012).

## Reagents

**Table:** 

**MUTANT**	**GENOTYPE**	**DEFECTS**	**AVAILABLE FROM**
*pf23-1*	*pf23* (without or with the *twi1* background)	Cytoplasmic preassembly of several ciliary dyneins (Huang et al., 1979; Yamamoto et al., 2017; Yamamoto et al., 2020)	*Chlamydomonas* resource center (https://www.chlamycollection.org/)
